# Development of a Carbon Nanotube-Based Touchscreen Capable of Multi-Touch and Multi-Force Sensing

**DOI:** 10.3390/s151128732

**Published:** 2015-11-13

**Authors:** Wonhyo Kim, Haekwan Oh, Yeonhwa Kwak, Kwangbum Park, Byeong-Kwon Ju, Kunnyun Kim

**Affiliations:** 1 Next Generation Convergence Sensor Research Center, Korea Electronics Technology Institute, 25, Saenari-ro, Bundang-gu, Seongnam-si, Gyeonggi-do 463-816, Korea; E-Mails: kimwh@keti.re.kr (W.K.); ajounasa@gmail.com (H.O.); yhkwak@keti.re.kr (Y.K.); parkkb@keti.re.kr (K.P.); 2 Display and Nanosystem Laboratory, Department of Electrical Engineering, Korea University, Anam-dong, Seongbuk-gu, Seoul 136-701, Korea

**Keywords:** single-walled carbon nanotubes (SWCNTs), touchscreen, multi-touch, multi-force

## Abstract

A force sensing touchscreen, which detects touch point and touch force simultaneously by sensing a change in electric capacitance, was designed and fabricated. It was made with single-walled carbon nanotubes (SWCNTs) which have better mechanical and chemical characteristics than the indium-tin-oxide transparent electrodes used in most contemporary touchscreen devices. The SWCNTs, with a transmittance of about 85% and electric conductivity of 400 Ω per square; were coated and patterned on glass and polyethyleneterephthalate (PET) film substrates. The constructed force sensing touchscreen has a total size and thickness of 62 mm × 100 mm × 1.4 mm, and is composed of 11 driving line and 19 receiving line channels. The gap between the channels was designed to be 20 µm, taking visibility into consideration, and patterned by a photolithography and plasma etching processes. The mutual capacitance formed by the upper and lower transparent electrodes was initially about 2.8 pF and, on applying a 500 gf force with a 3 mm diameter tip, it showed a 25% capacitance variation. Furthermore, the touchscreen can detect multiple touches and forces simultaneously and is unaffected by touch material characteristics, such as conductance or non-conductance.

## 1. Introduction

As mobile device usage has grown rapidly, so has interest in touchscreen input devices. Earlier research concentrated on push-button input which was used in a variety of industries and applications, such as robots, displays, and information technology fields. However, the transparent touchscreen has recently become widely adopted because of its increased simplicity and ease of use. The touchscreen can recognize touch position by simple contact and perform various functions by double-click motion.

Their touch-sensing mechanisms can generally be divided into four types, *i.e.*, resistive, capacitive, infrared, and acoustic [1,2,3,4,5,6,7,8,9]. The capacitive sensing type is widely used in mobile devices, such as smartphones and tablets, due to considerations such as fabrication costs, motion accuracy, and durability. However, despite the convenience of the touchscreen, the demand for more intuitive and realistic input devices has increased and there is a need for touchscreen devices that can simultaneously recognize not only the position but also the force of the touch.

**Figure 1:**
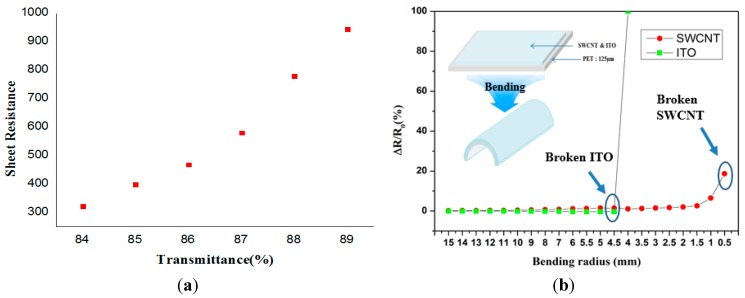
The sheet resistance graph for a 125 um thick PET film showing the transmittance and measured resistance change of an SWCNT electrode according to bending radius; The SWCNT electrode maintains a fairly stable resistance up to a bending radius of about 3 mm. (**a**) The measured sheet resistance and transmittance of coated SWCNTs; (**b**) The measured resistance change of SWCNTs and ITO electrode on the PET film according to bending radius.

Currently transparent electrodes made of indium oxide (ITO) doped with tin (In_2_O_3_:Sn) are typically used in touchscreens and optoelectronic devices because of their high transparency and low resistivity characteristics. However, the force sensing touchscreen proposed in this research, rather than ITO-based material, uses single-walled carbon nanotubes (SWCNTs) as transparent electrodes. This has three main advantages: first, the proposed touchscreen device can simultaneously measure both touch position and touch force, regardless of the type of touch media used. Secondly, it can maintain stability when subjected to stringent environmental conditions and external shocks [10,11]. Finally, compared to an ITO electrode, it has better mechanical and chemical properties. The most important mechanical property of the proposed device is its physical deformation properties, which we analyzed and compared to those of ITO though bending experiments. As an ITO transparent electrode is vulnerable to bending deformation, its use as a transparent electrode is limited to rigid devices. SWCNTs, however, can additionally be applied to flexible devices due to their superior deformation stability, as shown in Figure 1 [12].

## 2. Design and Fabrication

The proposed force sensing touchscreen is primarily composed of an upper/lower SWCNT transparent electrode substrate and force sensing layer, which can be easily transformed and restored by external loads. Each substrate consists of rows and columns electrodes assembled to form a matrix-type force sensing touchscreen. The lower transparent SWCNTs, which are on a glass substrate, and the upper transparent SWCNTs, which are on a PET film substrate, face each other. Between these two electrode substrates sits the transparent and elastic silicone gel, which acts as a force sensing layer. This layer creates a uniform electric capacitance between the two transparent electrodes, which varies according to the thickness of the force sensing layer. The increase in electric capacitance due to external loads altering the thickness of the force sensing layer between the electrodes is used to determine the touch position and the touch force. Although existing touchscreens only respond to the touch of a conductive material, usually a human finger, the force sensing touchscreen lacks such a restriction.

**Figure 2:**
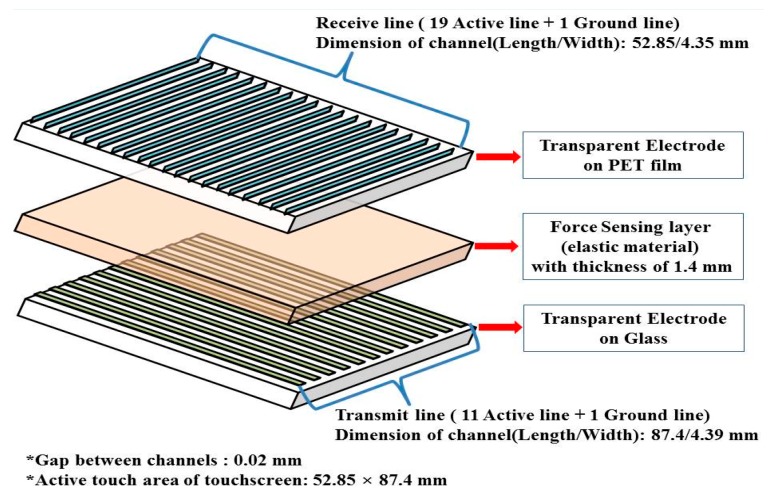
Schematic diagram of the force sensing touchscreen. Force sensing touchscreen is primarily composed of two patterned transparent electrode substrates and a force sensing layer with the property of elastic recovery.

Figure 2 shows a schematic diagram and the main design parameters of the force sensing touchscreen fabricated using SWCNTs. The total size of the proposed SWCNT force sensing touchscreen is 4 square inches of actual touch area, 52.85 mm wide and 87.4 mm long. The upper and lower transparent electrodes were designed to a rectangular form and composed of 19 receiving electrode and 11 transmitting electrode channels (Table 1). In order to maintain electric capacitance and recognize touch position and force through sensing changes in electric capacitance induced by the deformation of the distance between the two electrodes, the upper and lower electrode substrates of the rectangular form were assembled in a cross. For good visibility characteristics, the design of the force sensing touchscreen included a 20 µm gap between the electrode channels created using a micro-fine patterning process, and a 500 µm silicone gel material force sensing layer between the two electrode layers using an injection process. The elastic force sensing layer maintains an electric capacitance which remains constant in the layer’s neutral state but which changes as the layer is deformed by an applied force.

One of the most crucial stages in the fabrication of the capacitive force sensing touchscreen was the micro-patterning of the SWCNTs electrode. Typically, the SWCNT patterning process is not easy and is partially performed using a chemical etchant. However, this chemical etching process has the potential to damage the device and be toxic to the human body [13,14]. Therefore, many research groups have been studying the application of only a simple electrode which removes the need for the micro-patterning process. In this study, however, the force sensing touchscreen was successfully fabricated through SWCNT micro-patterning using a plasma etching process.

**Table 1:** Design parameters of force sensing touchscreen.

Item	Values	
The Total Size of the Force Sensing Touchscreen	62 mm × 100 mm	
Touch Area of Force Sensing Touchscreen	52.85 mm × 87.4 mm	
Number of Channel	X channel (Receive line)	20
	Y channel (Transmit line)	12
Dimension of Channel (Length/Width)	X channel (Receive line)	52.85 mm/4.35 mm
	Y channel (Transmit line)	87.4 mm/4.39 mm
Dimension of Trace Line (Width/Gap)		0.1 mm/0.1 mm
Gap between Channels		0.02 mm
Thickness of Force Sensing Touchscreen		1.4 mm

**Figure 3:**
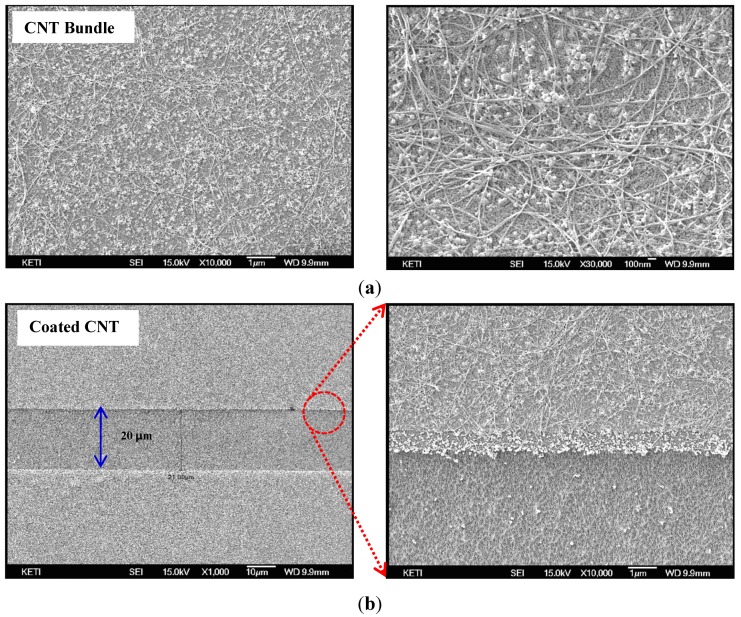
SEM images of micro-patterned SWCNTs on substrate; (**a**) SWCNTs like tangled hair bundles on a substrate after the spray coating process; (**b**) Coated and patterned SWCNT electrodes on a PET film and a glass substrate with a 20 μm gap between channels.

As shown in Figure 3, the SWCNTs appear as tangled hair-like bundles of about 100 µm in length and ~20 nm in size. Unneeded sections are removed by the plasma etching process. Since SWCNTs are made of carbon, under plasma treatment they react with oxygen to form carbon dioxide. By using oxygen plasma to burn away the carbon in a controlled manner so as to remove unwanted SWCNTs, the desired pattern of SWCNTs was left behind.

The fabrication process of the force sensing touchscreen consisted mainly of the formation of the upper and lower electrodes with SWCNTs, the assembly of the two electrodes, and the insertion of a force sensing layer. The transparent SWCNT electrode was coated on the substrate using a spray-coating method. Arc-discharging produced and thermally purified SWCNT was used as the transparent electrode material. SWCNTs were dispersed and chopped in deionized (DI) water by an ultrasound vibration process with the aid of surfactant and sodium dodecylbenzenesulfonate. The surfactants and sodium dodecylbenzenesulfonate were used to apply a uniform coat of SWCNTs to the substrate. The dispersed SWCNT coating solution contained 0.05 wt% of SWCNTs and 0.5 wt% of sodium dodecylbenzenesulfonate. After ultrasound treatment, the chopped SWCNTs were separated by weight and refined by a centrifugal system for pure refining. This process removed most of the impurities and the bundles of SWCNTs that had not been broken into small pieces. The transparent electrode coating process created an SWCNT coat of about 400 Ω electric sheet resistance and 85% transmittance at a wavelength of 550 nm. The coating process was performed on a hot plate, which was kept at 90 °C in order to remove the DI water medium in which the SWCNTs were dispersed for spraying (NDH-500, Nippon Denshoku Ltd., Tokyo, Japan).

The patterned SWCNT electrodes on the upper PET substrate have a rectangular geometry 4.35 mm in width and 52.85 mm in length and those on the lower glass substrate a rectangular geometry 4.39 mm in width and 87.4 mm in length. In addition, they had electrical resistances of about 6 kΩ and 9 kΩ on the upper and lower substrates, respectively. After completing the patterning process of the upper and lower electrode substrates, the flexible printed circuit (FPC) bonding process was carried out, followed by the silicone gel injection process which formed the force sensing layer of the touchscreen. The FPC for signal processing was bonded using anisotropic conductive film (ACF) made from circular nickel particles 6 μm in diameter. The last fabrication process, the injection of the compressive-elastic dielectric gel material (3-4154, Dow Corning, Midland, MI, USA) was achieved using an air pressure injector consisting of a metal needle and an air pressure controller. As silicone gel in solution has some viscosity, a 500 µm thick polycarbonate dam structure was attached to the edges of the electrode substrates before the injection process to prevent the silicone gel from leaking outside. After the injection process, the injected silicone gel was left at room temperature for approximately 12 h to solidify and gain its elastic property. The resulting force sensing touchscreen panel module had a type of sandwich structure.

## 3. The Experimental Results

**Figure 4:**
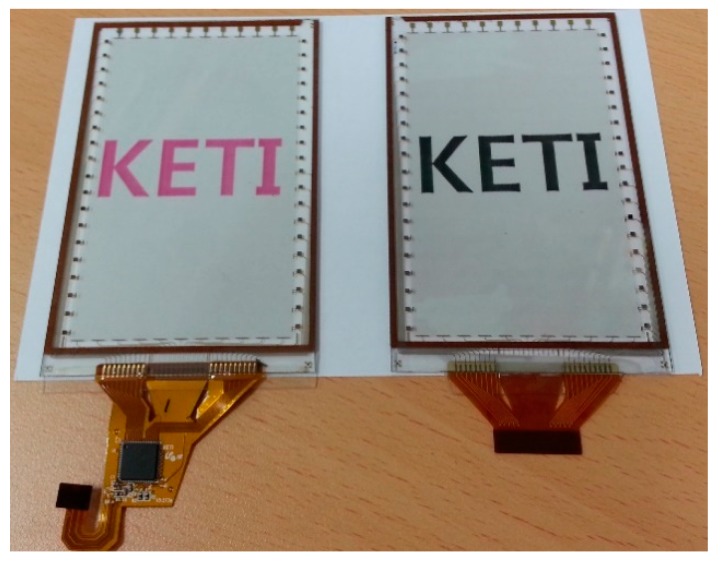
Image of the fabricated SWCNT based force sensing touchscreen composed of substrates of transparent glass and PET film and a force sensing inner layer of elastic silicone inserted between the two electrodes.

As the fabricated force sensing touchscreen (Figure 4) uses a capacitive-type method, it was important to characterize the capacitance changes caused by touch and touch force. The force sensing touchscreen maintained a uniform mutual capacitance at a regular distance between the two electrodes, and sensed touch position and touch force simultaneously by detecting changes in mutual capacitance caused by the applied force.

The force sensing touchscreen was composed of an 11 X-channel electrode and a 19 Y-channel electrode so that the total 209 mutual capacitances formed by the channel electrodes could be measured by capacitance measuring apparatus.

**Figure 5:**
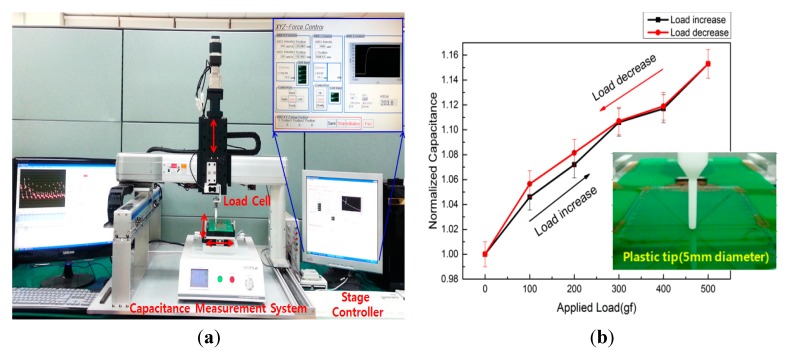
The system used to evaluate the force sensing touch screen and a graph of mutual capacitance variation against the force applied; (**a**) The evaluation system is composed of capacitance measurement equipment, a load cell for detecting the applied force and a stage controller for setting the location of the applied force on the sensing touchscreen; (**b**) A graph of mutual capacitance variation against applied force.

Figure 5 shows the system used to evaluate the force sensing touchscreen and a graph of variation in mutual capacitance variation against applied force. It mainly consists of capacitance measurement apparatus, the shaft connected to the load cell for measuring the applied force, and an X-Y stage for controlling the shaft’s position. The fabricated force sensing touchscreen was connected to the capacitance measurement apparatus using a flexible cable and the circular touch tip attached to the shaft was moved up, down, left, and right precisely. Translocating the circular tip in a vertically at the measurement position allowed the changes in capacitance output corresponding to changes in touch pressure to be measured. Tips from 3 mm to 10 mm in circular cross-section diameter and made of Teflon, were used to when determining the effects of the applied force which varied from 0 gf to 500 gf and in the reverse direction. 

**Figure 6:**
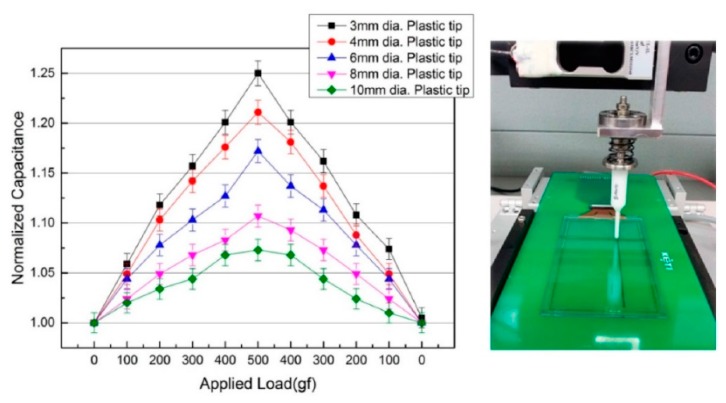
Graph of changes in capacitance value depending on the cross-sectional area of the tips used to apply the force to the center of the force sensing touchscreen; tips 3 mm to 10 mm in circular cross-sectional diameter were used to measure the effects of an applied force from 0 gf to 500 gf and in the reverse direction is also measured. (**Left**) The measured capacitance outputs according to circular cross-sectional diameter of the tip; (**Right**) The image of measuring output characteristics using the capacitance measurement system.

Figure 6 is a graph showing changes in capacitance depending on the size of the cross-sectional area of the tips applied to the center of the force sensing touchscreen: the smaller the size of the tip cross-sectional area, the greater the pressure at the constant force. The resulting large contractions of the force sensing layer between the two electrodes led to correspondingly large changes in capacitance due to the reduced distances between the two electrodes. The mutual capacitance under a 500 gf force applied with 3 mm diameter tip was measured to be approximately 2.8 pF on average representing a 25% variation in capacitance. As shown by the graph in Figure 6, with an applied force of 500 gf changes in capacitance led to relatively linear output characteristics and a small offset value for initial capacitance.

**Figure 7:**
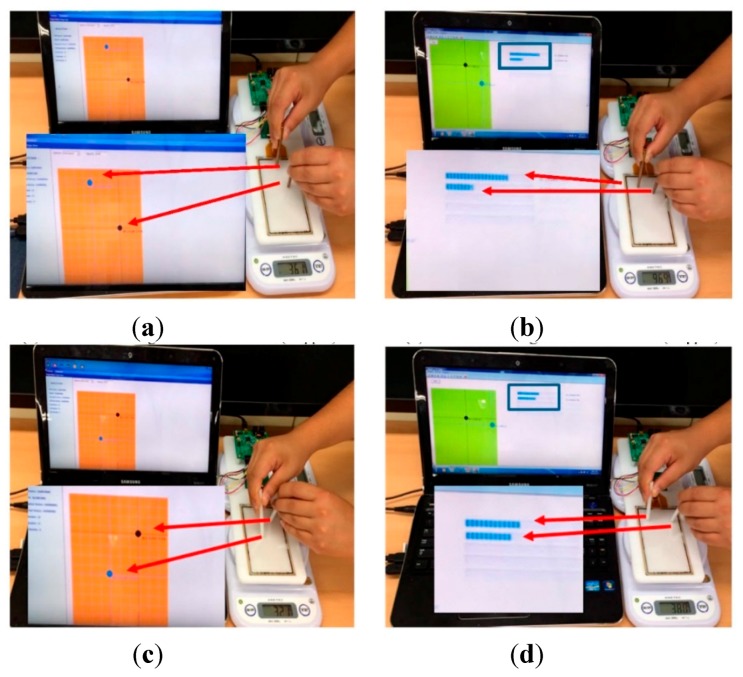
Operational performance of the force sensing touchscreen which was connected to the electrical board; (**a**) Multi-touch using conductor material (Copper); (**b**) Multi-force using conductor material (Copper); (**c**) Multi-touch using non-conductor material (Teflon) and (**d**) Multi-force using non-conductor material (Teflon) were displayed on the touch awareness program developed. The black and blue points represent touch positions and the intensity of the applied touch force was displayed by the movement of the scale bar (Arbitrary unit).

Another important characteristic of the force sensing touchscreen was the simultaneous detection of both multi-touch and multi-force. As shown in Figure 7, a normal force applied by either conductor and or a non-conductor of hemispherical shape generated a change in the distance between electrodes, and as a result, a variation in mutual capacitances was displayed as the touch position. The intensity of the applied force was also represented by the movement of the scale bar in the upper right. Although the measurement of multi-touch and the multi-force at just two points is shown in Figure 6, it is equally possible to measure more than two because all the mutual capacitances formed by the row and column electrodes were operated independently.

Conventional capacitive touchscreen only reacts to conductive touch such as that of a human finger. However, the force sensing touchscreen also operates with insulating materials, such as Teflon as used in our study. Indeed, the advantage of the force sensing touchscreen is that there are no restrictions on the type of touch material which can be used.

## 4. Conclusions

A force sensing touchscreen, which is capable of multi-touch and multi-force sensing, was designed and fabricated using SWCNTs as an alternative transparent electrode to conventional ITO-based electrodes. The SWCNT electrodes proved superior to ITO electrodes in their mechanical and chemical characteristics, and have excellent robustness to external environment and shock. The force sensing touchscreen was fabricated with an area of 4 square inches and a thickness of 1.5 mm. It consists of upper and lower transparent electrodes and maintains a uniform mutual capacitance. It shows relatively linear characteristics and has a 25% capacitance variation under a 500 gf force applied with a 3 mm diameter tip. It perceives the touch position and touch force by sensing the simultaneously resulting change in mutual capacitance. The SWCNTs were patterned using plasma etching technology with a gas mixture of oxygen and argon. The micro-patterning process was successfully carried out with a 20 µm gap between the SWCNT electrode channels. The force sensing touchscreen, with its two electrodes and dielectric material, has no restrictions with respect to the conductance or non-conductance of the touch material.

The force sensing touchscreen goes beyond the capabilities of existing touchscreens in that it not only recognizes the touch position but can also provide multi-tasking capabilities through simultaneous multi-touch and multi-force sensing. It is therefore applicable to diverse applications, such as mobile devices and flexible display fields that require touch devices and transparent electrodes.

## References

[1] Wen Y.C., Heng J.L. (2011). Real multitouch panel without ghost points based on resistive patterning. J. Disp. Technol..

[2] Ruan J.-Y., Chao P.C.-P., Chen W.-D. A multi-touch interface circuit for a large-sized capacitive touch panel. Proceedings of the 2010 IEEE Sensors.

[3] Lee J., Cole M., Lai J.C.S., Nathan A. (2014). An analysis of electrode patterns in capacitive touch screen panels. J. Disp. Technol..

[4] Chenchi L., Borkar M.A., Redfern A.J., MaClellan J.H. (2012). Compressive sensing for sparse touches on capacitive touch screens. IEEE J. Emerg. Sel. Top. Circuits Syst..

[5] Takasaki M., Nara T., Tachi S., Higuchi T. A tactile display using surface acoustic wave. Proceedings of the IEEE International Workshop on Robot and Human Interactive Communication.

[6] Kyu T.S., Hsu-hung H., Lee C.C. Generation of surface acoustic waves for general sensing applications. Proceedings of the 58th Electronic Components and Technology Conference (ECTC).

[7] Min M.A., Eou S.C., Sang J.K. (2014). Characteristics of ITO-resistive touch film deposited on a PET substrate by in-line DC magnetron sputtering. Vacuum.

[8] Sierros K.A., Morris N.J., Kukureka S.N., Cairins D.R. (2009). Dry and wet sliding wear of ITO-coated PET components used in flexible optoelectronic applications. Wear.

[9] Kim K.N., Shin K.W., Han J.H., Lee K.R., Kim W.H., Park K.B., Ju B.K., Park J.H. (2011). Deformable single wall carbon nanotube electrode for transparent tactile touch screen. Electron. Lett..

[10] Park J.M., Wang Z.J., Kwon D.J., Gu G.Y., Lawrence DeVries K. (2013). Electrical properties of transparent CNT and ITO coatings on PET substrate including nano-structural aspects. Solid State Electron..

[11] Ali L.N., Satoshi Y., Kazufumi K., Takeo Y., Don N.F., Hiroaki H., Motoo Y., Sumio L., Kenji H. (2010). Extracting the Full Potential of Single-Walled Carbon Nanotubes as Durable Supercapacitor Electrodes Operable at 4 V with High Power and Energy Density. Adv. Mater..

[12] Jung D.W., Lee K.H., Kim D.H., Dorothea B., Lawrence J.O., Lee G.S. (2013). Highly conductive flexible multi-walled carbon nanotube sheet films for transparent touch screen. Jpn. J. Appl. Phys..

[13] Kim J.T., Song M.J., Lee C.J., Lee J.H., Kim J.H., Min N.K. (2013). A comparative study of electrochemical and biointerfacial properties of acid-and plasma-treated single-walled carbon-nanotube-film electrode systems for use in biosensors. Carbon.

[14] Yang S., Songfeng P., Jinhong D., Wen B.L., Hui M.C. (2013). Patterning flexible single-walled carbon nanotube thin films by an ozone gas exposure method. Carbon.

